# Letter from the Editor in Chief

**DOI:** 10.19102/icrm.2018.091109

**Published:** 2018-11-15

**Authors:** Moussa Mansour


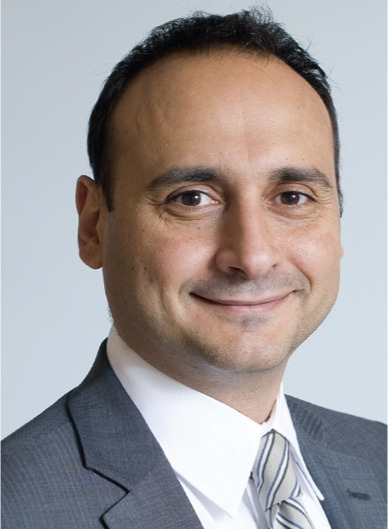


Dear Readers,

This issue of *The Journal of Innovations in Cardiac Rhythm Management* contains an important article by Hylind et al.^1^ titled “Genetic Testing for Inherited Cardiac Arrhythmias: Current State-of-the-Art and Future Avenues.” In it, the authors discuss the basic genetic concepts underlying long QT syndrome (LQTS) and catecholaminergic polymorphic ventricular tachycardia (CPVT), two arrhythmias with well-defined genetic mechanisms. They furthermore describe the predictive value of genetic testing and, most importantly, review genotype-guided treatment strategies.

Genetic testing as a means to better understand patients with inherited arrhythmias has become widely used. Many centers now have inherited arrhythmia clinics, with genetic counselors and physicians working closely together to provide patients and their families with individualized data regarding the possible onset of various cardiac diseases generated from the results of genetic testing as well as risk assessment services for family members and, to a lesser degree, guidance for treatment strategies. These facilities are not limited only to evaluating arrhythmias with well-defined genetic mechanisms such as LQTS and CPVT; they also strive to elucidate the genetic underpinnings of other cardiac conditions with less clear mechanisms, such as inherited cardiomyopathies and other arrhythmias that can cause sudden cardiac death.

One of the limitations of genetic testing, however, is the existing variation in the degree of the penetrance and expressivity of a gene mutation in affected individuals. The clinical presentation of a certain condition is often not just determined by a patient’s genetic profile: other factors such as environmental influences may also play a part. As a result, the role and success of genetic testing in the formulation of treatment plans remain limited in scope. Still, with the ongoing advances in technology continually allowing for a faster, more detailed, and increasingly accessible analysis of the genetic profile, it is perhaps foreseeable that clinical studies where genetic testing is investigated not only as a diagnostic tool but also as a major determinant of the treatment plan may exist in the near future.

I hope that you find the article by Hylind et al. and the rest of this issue educational and of value in your practices.

Sincerely,


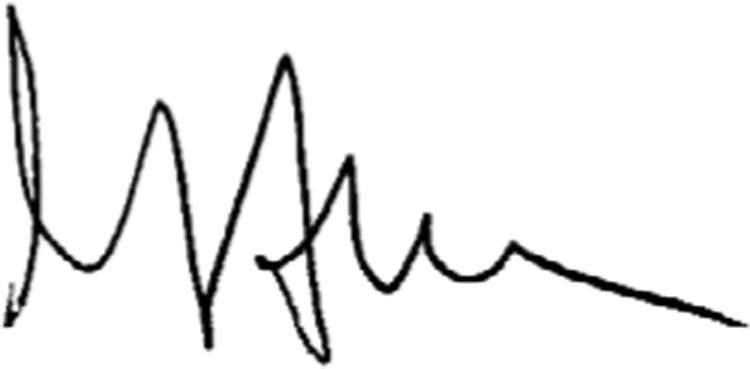


Moussa Mansour, md, fhrs, facc

Editor in Chief

The Journal of Innovations in Cardiac Rhythm Management

MMansour@InnovationsInCRM.com

Director, Atrial Fibrillation Program

Jeremy Ruskin and Dan Starks Endowed Chair in Cardiology

Massachusetts General Hospital

Boston, MA 02114
